# Supplementary material for: Patterns of Intron Gain and Loss in Fungi
Source: PLoS Biol. 2004 Nov 30;2(12):e422. doi: 10.1371/journal.pbio.0020422 (PMC532390; doi:10.1371/journal.pbio.0020422)
Supplement: Table S1 — Also available at http://genes.mit.edu/NielsenEtAl/. (4.3 MB ZIP). [file pbio.0020422.st001.zip › NielsenEtAl/html/729.html]

AN8637.1.NCU08791.1.MG10061.1.FG06554.1


```
 CLUSTAL W (1.82) Multiple Sequence Alignments - Introns Inserted


Sequence 1: MG10061.1	743 aa
Sequence 2: FG06554.1	738 aa
Sequence 3: NCU08791.1	765 aa
Sequence 4: AN8637.1	744 aa
Alignment Length: 778 aa
Number Identitical Residues: 368 aa
Alignment Score (without introns) 18781


MG10061.1 	---------------------------MASLKDIGDTLSKGKSYVMHS--QGDKISDLAR
NCU08791.1	MQRLALHYPPLDPAAEPPTEHILQQSNPTMSNIISQAGQKAKEALTSAP-SSKKVDDLKN
FG06554.1 	--------------------------------MTEQVTGAIKHAVMGH--KNDKIDQLKA
AN8637.1  	-----------------------------MATSITAGLQKAQQAVQDTATKNKKIVDISH
          	                               .         :  :   .:...*: ::  

MG10061.1 	NTVTPDEKGRQTTDAGVRITNPDDWLRVTSNDQIGPSLLEDQIAREK~IHRFDHERIPER
NCU08791.1	EFKETDKSARLTTDYGVKQTTADDWLRIVSDDKIGPSLLEDPFARER0IMRFDHERIPER
FG06554.1 	NMVEPNENSRITSDYGVKQNNTDQWLRVASEDQTGPSLLEDAFGREK0IHRFDHERIPER
AN8637.1  	DTVNVHTDQEQRTDFGVAITDPDHWLRVTNETHSGPSLLEDHIARER~IHRFDHERIPER
          	:    . . .  :* **  . .*.***:..: : ******* :.**: * **********

MG10061.1 	VVHARGTAAHGKFTLHKSIEHLTTAGVLTDTSRETPVFLRFSTVLGSRGSADTVRDVRGF
NCU08791.1	VVHARGSGAFGKFKVYESASDLTMAPVLTDTSRETPVFVRFSTVLGSRGSADTVRDVRGF
FG06554.1 	VVHARGAGAHGTFRLFESAADVTKAGLFTDTSRETPVFVRFSTVLGSRGSADTVRDVRGF
AN8637.1  	VVHARGTGAYGNFTLKESIEDLTYAGVLTDTSRNTPVFVRFSTVQGSRGSADTVRDVRGF
          	******:.*.*.* : :*  .:* * ::*****:****:***** ***************

MG10061.1 	AVKFYTPEG~NWDIVGNNIPVFFIQDSMKFPDVI~HAGKPEPDNEVPQAQSAHNNFWDFM
NCU08791.1	AVKFYTEEG~NWDLVGNNIPVFFIQDAIKFPDVI~HAGKPEPHNEVPQAQSAHNNFWDFQ
FG06554.1 	AIKFYTQEG1NFDIPGNNIPVFFIQDAMKFPDVI~HAGKPEPNNEVPQAQTAHNNFWDFM
AN8637.1  	AVKFYTDEG~NWDIVGNNIPVFFIQDAIKFPDFV1HAVKPEPHNEVPQAQTAHNNFWDFV
          	*:**** ** *:*: ***********::****.: ** ****.*******:******** 

MG10061.1 	YQHSEATHMFMWAMSDRTIPRSYRMMQGFGVNTYTLINAKGERHFVKFHFTPELGVHSLV
NCU08791.1	FNHTEATHMFTWAMSDRAIPRSLRMMQGFGVNTYTLINAQGKRHFVKFHWTPELGVHSLV
FG06554.1 	FEHPEATHMHTWAMSDRTIPRSYRMMQGFGVNTYTLVNEKGERRFVKFHWTPELGVHSLV
AN8637.1  	YLHPEATHMFMWAMSDRAIPRSYRMMQGFGVNTFSLVNKEGKRHFVKFHWIPHLGVHSLV
          	: *.*****. ******:**** **********::*:* :*:*:*****: *.*******

MG10061.1 	WDEALKLAGQDPDFHRKDLMEAIANGAFPRWKFGVQVLPESREHEFDFDILDATKVWPED
NCU08791.1	WDEALKLAGQDPDFHRKDLWEAIENGAYPKWKFGIQAIAEEDEHKFDFDILDATKIWPED
FG06554.1 	WDEALKLAGQDPDFHRKDLQEAIENGAFPKWKFGIQVLDESQEHDFDFDILDATKVWPED
AN8637.1  	WDEALKLAGQDPDFHRKDLMEAIDNKAYPKWDFAIQAIPEEDQDKFEFDIFDATKVWPEE
          	******************* *** * *:*:*.*.:*.: *. :..*:***:****:***:

MG10061.1 	LIPVDYIGTLELNRNIDEFFPETEQVAYCTSHVVPGIGFSDDPLLQGRNFSYFDTQISRL
NCU08791.1	LVPVRYIGEMELNRNPDEFFPQTEQIAFCTSHVVNGIGFSDDPLLQGRNFSYFDTQISRL
FG06554.1 	LIPVRYIGELELNQTVDEYFTQTEQVAFCTSHLVPGIGHSDDPLLQGRNFSYQDTQLSRL
AN8637.1  	QVPLRVVGELELNRNIDEFFPETEQVAFCTSHIVPGIDFSDDPLLQGRNFSYQDTQISRL
          	 :*:  :* :***:. **:*.:***:*:****:* **..************* ***:***

MG10061.1 	GINWEELPINRPVCPVLSFNRDGQSRHTITKGKVNYWPNRFGVNAPADGVKEGGYVEHAT
NCU08791.1	GVNFQELPINRPVCPVMNFNRDGAMRHTISRGTVNYYPNRFDACPPAS-LKEGGYLEYAQ
FG06554.1 	GTNWEELPINKPVCPVMNFNRDGAMRHTITKGKVNYWPNRFSHQPPAT-ESEGAYVDYAA
AN8637.1  	GVNWEEIPINRPVCPFLNHNRDGAKRHRITKGTVNYWPNRFEANPPAS---DKGFKSHPA
          	* *::*:***:****.:..****  ** *::*.***:****   .**    : .: .:. 

MG10061.1 	KVQGIKARTKSAKFLDHISQAQLFFNSMSDVEKRHMLAAFSFELDHCEEEVVYNNLV-SR
NCU08791.1	KVAGIKARARSAKFKEHFSQAQLFYNSMSPIEKQHMINAFGFELDHCEDPVVYGRMV-QR
FG06554.1 	KIVGMKQRTLSKKFKDHFTQAQLFYNSLSEIEKSHVQAAYSFELDHCDEAIVYERLT-ER
AN8637.1  	PITGRKRRDLTPKFKEYHNQAQLFYNSLSEVEKVHVKKAFSFELDHCDDPIVYERLAGQR
          	 : * * *  : ** :: .*****:**:* :** *:  *:.******:: :** .:...*

MG10061.1 	LTQIDIGLAQQVAEMVGAQIPDKATKENKGIKAKGLSQFDFLPENPTIASRKVAILIADG
NCU08791.1	LADIDLGLAQTIAEMVGGEAPTTTNHPNHGRKTINLSQTEFPPATPTIKSRRVAIIIADG
FG06554.1 	LGEIDGDLANTIAEMVGGKKPANP-KPNAGKKAKNLSQLDFVPETPTIKSRRIAIIIADG
AN8637.1  	LAEIDLPLAQAVAEMVGAPIPTKALRDNHGKTSVRLSQFDFTPKAPGIISRRIAIIIGDG
          	* :**  **: :*****.  * .. : * * .:  *** :* *  * * **::**:*.**

MG10061.1 	YDSVQFRAVKAALSAAQAIPLVIAPRRSPIYAAGETKGSSGAGLKPDHHLEGFRSTMVDA
NCU08791.1	YDNVAYDAAYAAISANQAIPLVIGPRRSKVTAANGS------TVQPHHHLEGFRSTMVDA
FG06554.1 	YDPVAFNAMYGAIKAQSALPFVIAPRRAPIFSAAED-SSSSKGIFPDHHLEGQRSTMFDA
AN8637.1  	YDKIAFNGMKAAILAAQALPFVIGTKRSAIYAQGED-KNSSKGVIPDHMYDGMRSTMFDA
          	** : : .  .*: * .*:*:**..:*: : :      .:.  : *.*  :* ****.**

MG10061.1 	VYVPGGAESIKTLSKNGRALHYVREAFGHLKAIGASGEGVELVKLAVT-LPEVTVSSAD-
NCU08791.1	IFIPGGAKAAETLSKNGRALHWIREAFGHLKAIGATGEAVDLVAKAIA-LPQVTVSSEA-
FG06554.1 	IFVPGGAKSIETLSKNGRALHYIREAFGHLKTIGGTGEAVEFINKAIQ-LSEVALSSTDG
AN8637.1  	TFIPGGS-HIETLQKNGQIRYWIAETFGHLKALGAMGEAAQLVKEVLGNVMGVQIAGADS
          	 ::***:   :**.***:  ::: *:*****::*. **..:::  .: .:  * ::.  .

MG10061.1 	--VMESYGVVTVRN-VQADSLKEVVDLAKGGKDFLDQFFHSIAMHRNWAREADGLNSQVA
NCU08791.1	-EVHESYGVVTLKK-VKPESFTDAVKIAKGAAGFLGEFFYAIAQHRNWDRELDGLHSMIA
FG06554.1 	SGVVDSYGVVTLKN-ASPDTLKEIATVASDAKGFLEKFVYNVSCHRNWQRELDGLSTMVA
AN8637.1  	AEPVEWYGVVTARGPESAESLSEGFKVLKDAGDFTSKFFYQISQHRNWQRELDGLASTVA
          	:   : ***** : . ..:::.:   : ... .*  :*.: :: **** ** *** : :*

MG10061.1 	Y
NCU08791.1	Y
FG06554.1 	Y
AN8637.1  	F
          	:
```
